# Can Attention Deficits Predict a Genotype? Isolate Attention Difficulties in a Boy with Klinefelter Syndrome Effectively Treated with Methylphenidate

**DOI:** 10.1155/2014/980401

**Published:** 2014-08-19

**Authors:** Antonella Gagliano, Eva Germanò, Loredana Benedetto, Gabriele Masi

**Affiliations:** ^1^Division of Child Neurology and Psychiatry, Department of Pediatrics, University of Messina, Via Consolare Valeria, 98125 Messina, Italy; ^2^Division of Psychology, Department of Humanities and Social Sciences, University of Messina, Via Concezione, No. 6/8, 98100 Messina, Italy; ^3^IRCCS Stella Maris, Scientific Institute Child Neurology and Psychiatry, Viale del Tirreno, No. 331, 56018 Calambrone, Pisa, Italy

## Abstract

This paper describes a 17-year-old boy who was diagnosed with Klinefelter syndrome (KS) (XXY) at the age of 16 years. Although cognitive level was absolutely normal, he showed attentional difficulties that negatively affected school adjustment. He was successfully treated with methylphenidate. A significant improvement was observed in the ADHD Rating Scale IV and in the inattention subscale score of the Conners Scales. The CGI-S score improved from 3 to 1, and the CGI-I score at the end point was 1 (very much improved). Also attention measures, particularly forward and backward digit span, improved with MPH treatment. Given the widely variable and often aspecific features, KS may run undiagnosed in a large majority of affected patients. A close attention to the cognitive phenotype may favour a correct diagnosis, and a timely treatment.

## 1. Introduction

Klinefelter syndrome (KS) (47, XXY) is a sex chromosome aneuploidy associated with speech and language deficits, socioemotional difficulties, motor dysfunction, and frontal lobe deficits including attention, planning, and organization, possibly in response to the pubertal hormonal abnormalities. It is the most common chromosome abnormality in humans (1 : 500 to 1 : 1000 males), but due to the widely variable and often aspecific features, only one out of four cases are recognized [1]. Some studies hypothesize that supernumerary X chromosome and/or congenital hypogonadism can favour structural alterations in the subcortical pathways involved in language processing, thus providing a neurobiological substrate for cognitive deficits in KS. The phenotype might be due to overexpression of genes on the extra X chromosome. Examination of X-linked differentially expressed genes, such as GTPBP6, TAF9L, and CXORF21, suggesting verbal cognition-gene expression correlations, may establish a causal link between these genes, neurodevelopment, and language function [2]. In order to explain the linguistic impairment, the neurexin-neuroligin hypothesis has been recently proposed [3]. Neuroligin genes, on both X and Y chromosomes, are involved in the same synaptic networks as neurexin genes, with common variants associated with increased risk for language impairment and autism. The effect of a triple dose of neuroligin gene product is particularly detrimental when associated with specific variants of neurexin genes on other chromosomes. Structural brain abnormalities have been also described by MRI, such as a decreased brain volume, particularly in frontal lobe, temporal lobe, and superior temporal gyrus were observed bilaterally in a sample of XXY men [4]. Cognitive phenotype is extremely etherogeneous. Youths with KS may present deficits in language skills, verbal processing speed, verbal and nonverbal executive abilities, motor dexterity, and in reading and spelling [5]. Early motor and speech disturbances are the earlier presentation of the central nervous system dysfunction associated with androgen deficiency that is influential in brain organization, neurobehavioral development, temperament, and mood [6]. Neuropsychological deficits have been also reported in tasks exploring executive functions (EF). Recent findings suggest that executive dysfunctions associated with KS can be selectively identified, and they are particularly evident in the inhibitory subcomponent [7]. The attention and behavioural features reported in KS boys, namely, the attentional deficits, are often consistent with a cooccurring diagnosis of attention deficit/hyperactivity disorder (ADHD) [8]. Behavioral features are not homogeneous, including attention disorders, impaired social skills, autism spectrum symptoms, and other psychiatric disturbance [9]. There is also a strong variability among affected individuals, from minimal to significant cognitive and behavioral disorders [10]. When patients with KS have a normal IQ, the attention deficit could be a strong indicator of a genotype that may be otherwise unrecognized. Moreover, during prepubertal age, pathognomonic clinical features of KS are often lacking, but a characteristic cognitive and behavioral pattern is usually evident [11]. Early detection and immediate starting of educational supports is crucial to ameliorate the outcome and to reduce the psychopathological risk. This paper describes and comments the case of a KS boy with normal cognitive abilities and selective attentional deficits, successfully treated with methylphenidate (MPH).

## 2. Case Presentation

L. is a 17-year-old boy who was diagnosed with KS (XXY) at the age of 16 years. His physical characteristics included tall stature, hypogonadism, and fertility problems. After an uneventful full-term birth, he had normal cognitive and motor development and only mild language delay, with rapid spontaneous normalization. During primary school, modest academic difficulties, but no academic failures, are reported. Emotional and social development was normal, with mild, not impairing social anxiety, subthreshold obsessive-compulsive symptoms and a mild weakening in self-esteem. Although cognitive level, assessed by Wechsler Intelligence Scale for Children (WISC-III) at 12 years old, was absolutely normal (Verbal IQ 108; Performance IQ 115; Full IQ 110); he showed attentional difficulties that negatively affected school adjustment. However, he was able to attend secondary school with no help. But, during his third level of junior high school, his difficulties grew and he failed a grade. Given the persisting attentional difficulties during the fourth year of junior high school, parents agreed to start treatment with methylphenidate immediate release (MPH). At that time L. was drug naïve and not treated with testosterone. MPH was started at a dose of 10 mg/day b.i.d. (0.3 mg/kg/day; weight 66 kg) (morning and early afternoon), with weekly increments with flexible dosing strategy of 5 mg for each administration, up to 40 mg/day b.i.d. (0.6 mg/kg/day). The followup was performed at baseline and at the end point of the 3rd month after the start of MPH. Behavioural assessment was performed according to parent- and self-report scales and was performed by attentional tasks. The primary measure of effectiveness was the ADHD-RS-IV [12], with 18 items, rated on a scale from 0 (never/rarely) to 3 (very often). Secondary outcome measures were Conners Rating Scale-Revised, Short Form for Parents (CPRS), Teachers (CTRS), and Youth (CY-self-report) [13] and Clinical Global Impressions-Severity (CGI-S) and Improvement (CGI-I) [14]. The CPRS is an assessment tool that provides valuable information about the child's behavior. This instrument is helpful when a diagnosis of ADHD is being considered and when follow-up measures are required. It consists of four distinct subscales: (1) oppositional (this subscale indicates an individual with a tendency to break rules and to have problems with persons in authority); (2) inattention (it specifies problems organizing own work, completing tasks on schoolwork, or concentrating on tasks that require sustained mental effort); (3) hyperactivity (this subscale indicates a subject having difficulty sitting still or remaining at the same task for very long; (4) ADHD index consists of the single best set of items for differentiating children/adolescents with attention problems from those without attention problems.

Emotional symptoms were evaluated using a self-report scale for depressive symptoms (Children's Depression Inventory, CDI) [15] and a self-report Multidimensional Anxiety Scale for Children (MASC) [16]. Diagnostic assessment included also electroencephalogram (EEG) and magnetic resonance imaging (MRI), that were both normal. We used the “Di Nuovo” attention test (DNAT) as assessment instrument to measure the attentive subdomains [17], a neurophysiological measure of attention, based on a computerized series of tasks that assess the responses to either visual or auditory stimuli. The DNAT indices include both visual and auditory information processing, omission and commission errors, and reaction times. Furthermore a short term memory task (forward and backward digit span), encompassed in DNAT, was assessed. All attention measures were assessed between the 2nd and the 3rd hour after the administration of the MPH. Response to treatment was evaluated according to changes from the baseline (pretreatment) to the end point at week 12 (posttreatment). Weight, height, blood pressure and pulse were evaluated at each visit. The study was approved by the local Ethics Committee.

**Figure 1:**
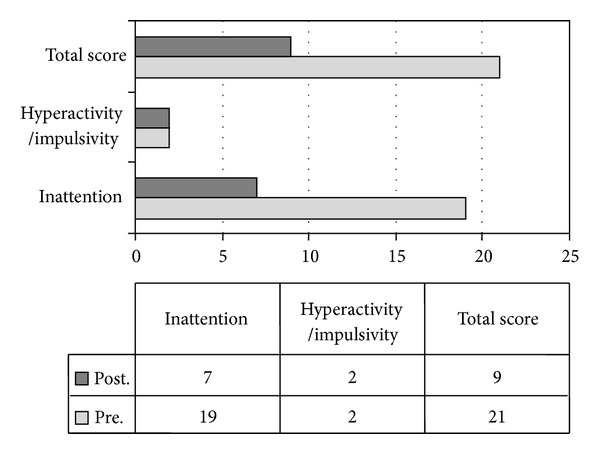
ADHD-RS-IV-Parent:Inv: raw scores in baseline (pretreatment) and with MPH treatment (posttreatment). The total score improved from 21 (*M* = 2.22, SD = 0.97) to 9 (*M* = 1.00, SD = 0.71), paired-sample *t*(8) = 5.50; *P* < .001, one-tail, and Cohen's *d* = 1.43.

A significant improvement was observed in the primary outcome measure (ADHD Rating Scale IV). The total score (rated and scored by investigators based on parent reports; ADHD-RS-IV-Parent:Inv) changed from 21 (*M* = 2.22, SD = 0.97) to 9 (*M* = 1.00, SD = 0.71), paired-sample *t*(8) = 5.50; *P* < .001, one-tail, and Cohen's *d* = 1.43 (Figure 1).

The CGI-S score improved as well from 3 to 1, and the CGI-I score at the end point was 1 (very much improved).

**Figure 2:**
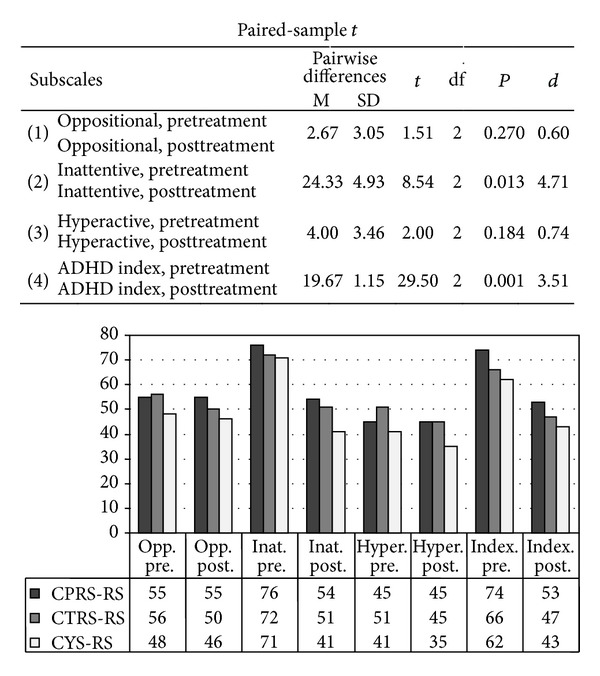
Conners Rating Scale for Parents (CPRS-RS), Teachers (CTRS-RS), and Boy (Conners Youth Self-report Rating Scale, CYS-RS); *T* scores in baseline (pretreatment) and with MPH treatment (posttreatment). Pairwise differences calculation has been performed between pre- and posttreatment scores. These scores corresponded to the mean values between parents, teachers, and self-report scores at CPRS, calculated for all four subscales: (1) oppositional, (2) inattentive, (3) hyperactive, and (4) ADHD index).

According to the secondary measures, the CPRS Inattention subscale of parents and teachers significantly improved as well as boy's ratings of CY-self-report. Pairwise differences calculation has been performed between pretreatment (*M*_pre_) and posttreatment scores (*M*_post_). These scores corresponded to the mean values between parents, teachers, and self-report scores at CPRS, calculated for all four subscales: (1) oppositional, (2) inattentive, (3) hyperactive, and (4) ADHD index; *M*_pre_ = 73.00 (SD_pre_ = 2.65) versus *M*_post_ = 48.67 (SD_post_ = 6.81), paired-sample *t*(2) = 8.54; *P* < .01, one-tail, and Cohen's *d* = 4.71 (Figure 2).

**Figure 3:**
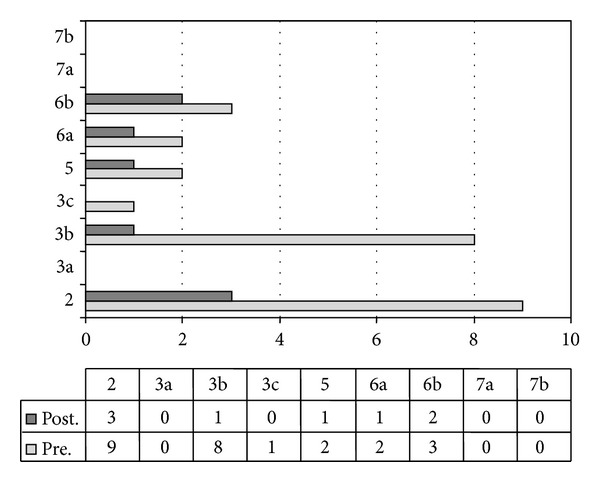
“Di Nuovo” attention test is the number of commission errors on nine attention tasks in baseline (pretreatment) and with MPH treatment (posttreatment). The mean value of all nine subscores pretreatment (*M*_pre_) was compared to the mean value of all nine subscores posttreatment (*M*_post_). *M*_pre_ = 2.78 (SD_pre_ = 3.42) versus *M*_post_ = 0.89 (SD_post_ = 1.05); nonparametric Wilcoxon test *z* = −2.26; *P* < .05, one-tail. Description of tasks is as follows: 2: multiple choice (visual stimuli); 3A: selective attention (auditory stimuli); 3B: selective attention (visual stimuli); 3C: barrage (visual stimuli); 5: divided attention; 6A: Stroop task-trial A; 6B: Stroop task-trial B; 7A: multiple barrage (auditory stimuli); 7B: multiple barrage (visual stimuli).

The attention tests (DNAT) at the baseline showed scores below the average in two attentional tasks (task 2: multiple choice of visual stimuli; task 3B: visual selective attention). All attentional scores significantly improved after MPH treatment. A comparison between the mean value of all nine subscores pretreatment (*M*_pre_) and the mean value of all nine subscores posttreatment (*M*_post_) was performed: *M*_pre_ = 2.78 (SD_pre_ = 3.42) versus *M*_post_ = 0.89 (SD_post_ = 1.05); nonparametric Wilcoxon test *z* = −2.26; *P* < .05, one-tail (Figure 3). All measures significantly improved.

**Figure 4:**
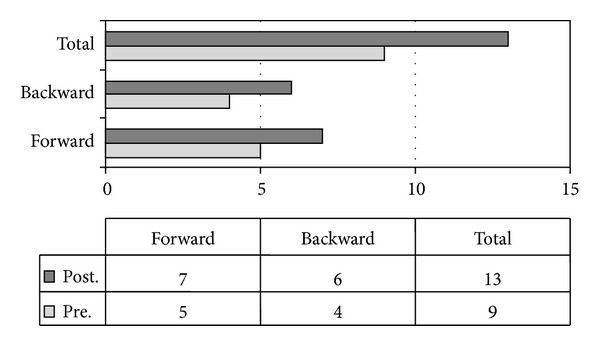
Digit span: number of forward and backward digits in baseline (pretreatment) and with MPH treatment (posttreatment).

The DNAT forward and backward digit span improved with MPH treatment (Figure 4).

In addition, over a three-month MPH treatment, parents and teachers reported strong improvement in academic performances, with upgrading of the evaluations in all the domains. At the baseline, no significant self-reported depressive symptoms (CDI score 11, below the cutoff score 19) were reported, but subtle anxiety symptoms were detected. At the baseline MASC, subtle anxiety symptoms were detected. After the treatment, basal MASC global score decreased from 54 to 45 and anxiety disorder index from 56 to 48, with the main effect in social anxiety dimension score. Neither adverse effects nor medication-related problems were reported.

## 3. Discussion

Compared to other genetic syndromes deriving from chromosomal trisomy, cognitive abilities in KS may be apparently normal, although a specific assessment may evidence more subtle cognitive and behavioral impairments affecting social, emotional, and academic functioning. This case report focuses on cognitive features in a boy with KS and comorbid ADHD, inattentive subtype. Comorbid ADHD in males with XXY is frequent, and it may be strongly related to poorer EF skills [18]. More in general, deficits in the ability to sustain attention with or without impulsivity are frequently reported in young boys with KS, and they can represent a component of the KS cognitive phenotype [5]. Nevertheless, there is a lack of data in the literature on ADHD treatment in KS. A recent paper of Tartaglia and colleagues [8] shows that psychopharmacologic treatment of ADHD with stimulants was effective in 73% of XXY, with a relatively low rate of significant side effects. Moreover, KS increased vulnerability to psychiatric disorders, such as ADHD, and to difficulties in language skills and social interactions can reveal important insights into genotype-phenotype associations [19]. Persisting school difficulties are usual, even in patients with normal IQ, with special needs of educational support. An analysis of these associations can yield more insight into genotype-phenotype associations [19], with implications on treatment. Our patient shows scores below the average in two attentional tasks regarding visual multiple choice and divided-attention tasks. Both neuropsychological deficits and scholastic difficulties dramatically improved during MPH treatment. Both visual multiple choice and divided-attention tasks improved, and MPH was effective as in patients without KS. The improvement of his divided-attention ability with MPH treatment is consistent with the behavioural measures of attention capacities. Actually, improvement in visual attention can lead to a variety of changes in behavior, from more efficient information processing, to a large extent, what information about the environment is perceived. These abilities are conceptually related to working memory. Working memory span tasks may also measure interference proneness and suggests that resistance to interference may affect performance on many cognitive tasks. In our patient, the verbal working memory, as measured by performance on the backward digit span task, seems to be improved by MPH treatment. This evidence is consistent with a recent meta-analysis on effects of MPH on cognitive functions in children and adolescents with ADHD [20]. Furthermore, MPH appeared helpful for anxious symptoms in our KS boy, as a consequence of positive changes in academic and social performances. This new condition could have positively influenced the emotional state, ameliorating his emotional symptoms and, subsequently, his enthusiasm and motivation to achieve scholastic contents. Our report, next to others that document psychiatric and social difficulties in KS patients, underlines that adaptive functioning is not only dependent on particular cognitive ability level, but also on the capability to use every skills effectively in order to get used to the social and work demands of everyday life. Given the pivotal role of attention in typically developing children in driving early developmental changes and outcomes and also more generally in shaping the broader sociocognitive landscape, some authors strongly suggest to extend the research to atypical populations, focusing on neurodevelopmental disorders with a clearly defined genetic origin [21]. Given the widely variable and often aspecific features, KS may run undiagnosed in a large majority of affected patients. A close attention to the cognitive phenotype may favour a correct diagnosis and a timely treatment. Psychiatric comorbidity in KS can be neglected as well. Symptoms of ADHD, and particularly attentional deficits, may be an important component of cognitive phenotype, even in patients with normal IQ. Thus it seems of paramount importance to explore how attention and other behavioral difficulties may constrain learning and sociocognitive outcomes across genetic neurodevelopmental disorders. When correctly diagnosed, ADHD in KS can be effectively treated with MPH across developmental time, even in late adolescence, and attentional deficits may strongly improve, with positive effects on academic performances and on emotional and social functioning.
